# Automatic Tissue Differentiation Based on Confocal Endomicroscopic Images for Intraoperative Guidance in Neurosurgery

**DOI:** 10.1155/2016/6183218

**Published:** 2016-04-05

**Authors:** Ali Kamen, Shanhui Sun, Shaohua Wan, Stefan Kluckner, Terrence Chen, Alexander M. Gigler, Elfriede Simon, Maximilian Fleischer, Mehreen Javed, Samira Daali, Alhadi Igressa, Patra Charalampaki

**Affiliations:** ^1^Siemens Healthcare, Technology Center, Princeton, NJ 08540, USA; ^2^Siemens Corporate Technology, 81739 Munich, Germany; ^3^Department of Neurosurgery, Hospital Merheim, Cologne Medical Center, 51109 Cologne, Germany; ^4^Department of Neurosurgery, Heinrich Heine University Düsseldorf, 40255 Düsseldorf, Germany

## Abstract

Diagnosis of tumor and definition of tumor borders intraoperatively using fast histopathology is often not sufficiently informative primarily due to tissue architecture alteration during sample preparation step. Confocal laser microscopy (CLE) provides microscopic information of tissue in real-time on cellular and subcellular levels, where tissue characterization is possible. One major challenge is to categorize these images reliably during the surgery as quickly as possible. To address this, we propose an automated tissue differentiation algorithm based on the machine learning concept. During a training phase, a large number of image frames with known tissue types are analyzed and the most discriminant image-based signatures for various tissue types are identified. During the procedure, the algorithm uses the learnt image features to assign a proper tissue type to the acquired image frame. We have verified this method on the example of two types of brain tumors: glioblastoma and meningioma. The algorithm was trained using 117 image sequences containing over 27 thousand images captured from more than 20 patients. We achieved an average cross validation accuracy of better than 83%. We believe this algorithm could be a useful component to an intraoperative pathology system for guiding the resection procedure based on cellular level information.

## 1. Introduction

Therapy of choice in most malignant and benign tumors in brain is to attempt a total resection with preservation of normal functional tissue, followed by radiochemotherapy. An incomplete resection of a tumor with remaining infiltrative growing cells increases the risk of recurrence with adjacent therapies, decreasing the quality of life and shortening lifetime.

The determination of tumors' borders during the operation is primarily based on the surgeon visual inspection on the tissue through microscope or a histopathologic examination of a limited number of biopsy specimens. Surgical pathology is focused on arriving at a definitive diagnosis of disease in the excised sample, involving a combination of gross, histologic, or even evaluation of molecular properties through immunohistochemistry. Unfortunately, intraoperative histopathology is often not sufficiently informative. Often the biopsies are nondiagnostic due to many reasons. These include sampling errors, which account for possible fact that the biopsies may not originate from the most aggressive part of the tumor. Furthermore, the tissue architecture of the tumor can be altered during the specimen preparation (e.g., frozen section [1]). Other disadvantages are the lack of interactivity and a waiting time of about 30–45 minutes for the result. The impact of “lower quality” slides on the diagnosis and surgical management is investigated in [1]. In summary, optimal surgical therapy, which is the combination of maximal near total resection and minimal injury of the normal tissue can only be achieved if the assessment of cellular, vascular, and connective structures to differentiate tumor from normal functional tissue is done accurately and during the course of operation with minimal time delay (preferably real-time).

**Figure 1:**
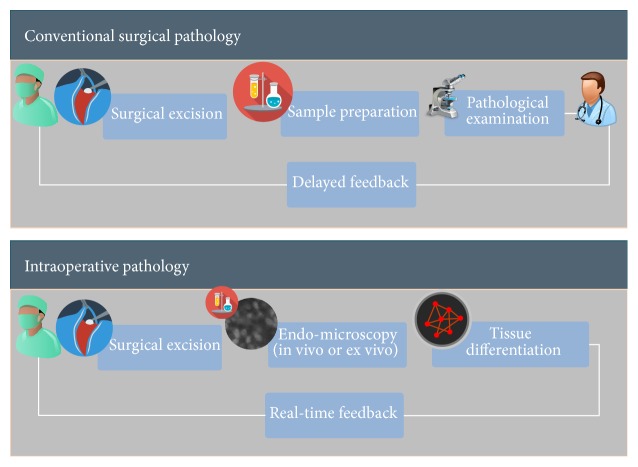
Conventional intraoperative pathology versus proposed intraoperative pathology.

**Figure 2:**
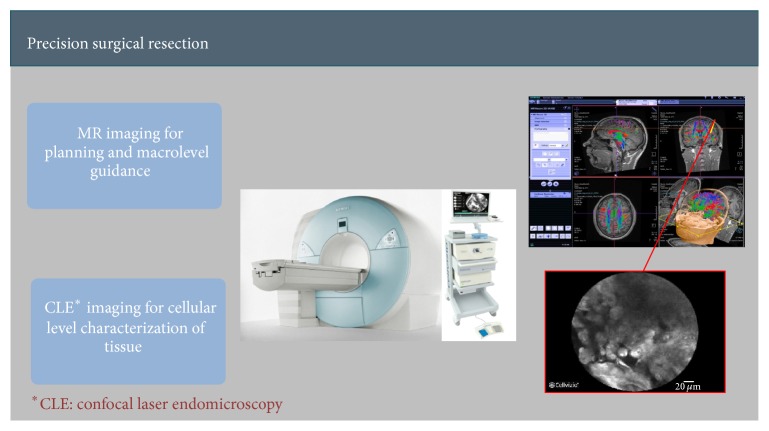
Potential application of intraoperative pathology and surgical guidance within a hybrid OR (this concept is an investigational tool and not approved for clinical use).

A number of recently introduced optical imaging technologies have started to be utilized in the clinical setting both macroscopically and microscopically during surgeries. Endomicroscopy is a technique for obtaining histology-like images from inside the human body in real-time [2], process known as “optical biopsy” [3]. It generally refers to fluorescence confocal microscopy, although multiphoton microscopy and optical coherence tomography have also been adapted for endoscopic use [4]. These images provide abundant information regarding cellular, vascular, and connective tissue structures and specific descriptors which could be used to differentiate various tissue types [5]. Our aim is to be able to computerize these analyses and assist surgeons in delineating tissue boundaries by analyzing real-time streams of images as quickly as possible (see Figures 1 and 2).

To this end, in this paper we propose a method for classifying cellular images and videos, which consist of a multistage image processing pipeline by extracting pixel level features and descriptors. We apply a learnt codebook to quantize each descriptor and generate specific code for each. An image or sequences of images are then represented by pooling result of generated codes into histograms. The pooling result is then used for classifying the image(s) contents into multiple types. The major novelty of our system includes (1) a processing pipeline, where a variety of the feature extraction methods, codebook generations, and classification algorithms can easily be tested and validated, (2) an advanced feature coding method that takes into account both locality and sparsity for computing feature codes, (3) a fast approximate optimization algorithm that significantly improves the computation efficiency with little compromise on ultimate system performance, (4) an image entropy-based data pruning function that increases system robustness to outliers, and (5) a majority voting based classification scheme that boosts the recognition performance for video stream based input data. Experiments demonstrate that the proposed system and method perform well on cellular image classification tasks.

## 2. Material and Methods

We use a commercially available clinical endomicroscope on the market from Mauna Kea Technologies, Paris, France, called Cellvizio. The main applications for such system are currently in imaging of the gastrointestinal tract, particularly for the diagnosis and characterization of Barrett's Esophagus, pancreatic cysts, and colorectal lesions. We use this system for imaging excised brain tissue during the operation for the identification of the tumor type. The image processing and classification pipeline is developed to automate the process of assigning labels to tumor types.

### 2.1. System Description

We use a commercially available clinical endomicroscope on the market Cellvizio (Mauna Kea Technologies, Paris, France). The main applications are currently in imaging of the gastrointestinal tract, particularly for the diagnosis and characterization of Barrett's Esophagus, pancreatic cysts, and colorectal lesions. Cellvizio is a probe-based CLE system. It consists of a laser scanning unit, proprietary software, a flat-panel display, and miniaturized fiber optic probes. The device is intended for imaging the internal microstructure of tissues in the anatomical tract (gastrointestinal or respiratory) that are accessed by an endoscope. Endomicroscopy enables subsurface analysis of the gut mucosa and in vivo histology during ongoing endoscopy in full resolution by point scanning laser fluorescence analysis [6]. Cellular, vascular, and connective structures can be seen in detail. The new detailed images seen with confocal laser endomicroscopy are unequivocally the beginning of a new era, where this optical development will allow a unique look onto cellular structures and functions at and below the surface of the gut. We consider the application of this technology for brain surgery where identification of malignant (glioblastoma) and benign (meningioma) tumors from normal tissues is clinically important.

### 2.2. Method Description

**Figure 3:**
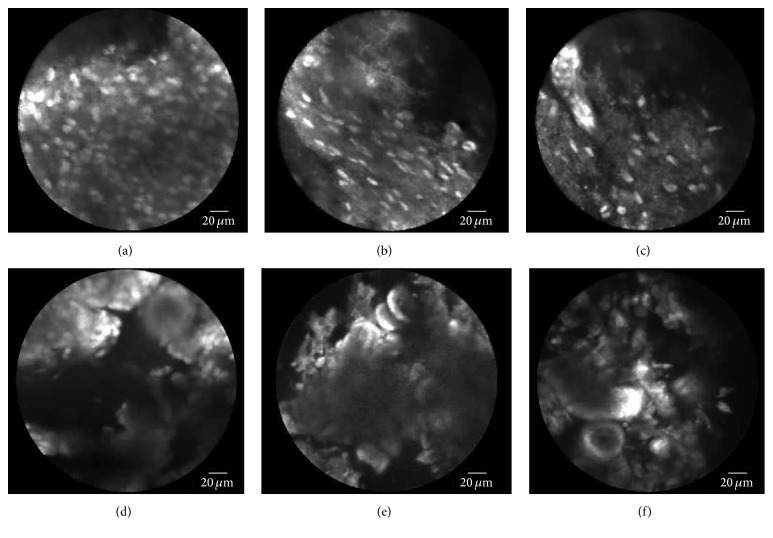
Examples of brain tumor imaging by endomicroscopy technology. (a)–(c) Glioblastoma (tumor) images. (d)–(f) Meningioma (tumor) images.

Specimens were collected from patients undergoing neurosurgical operations at the Department of Neurosurgery of the Hospital Merheim, Cologne Medical Center, in Cologne, Germany. All studies on human subjects were performed according to the requirements of the local ethic committee and in agreement with the Declaration of Helsinki. Tissue samples were excised from the tumor bed after the resection of the tumor. First, 1-2 drops of 0.01 mg/mL acriflavine hydrochloride AF from Sigma Pharmaceuticals, Victoria, Australia, dissolved in saline were administered topically to the excised tissue sample. This is primarily to stain the nuclei and to a minor extent the cell membrane and the extracellular matrix. The excess dye was washed off with saline. In this study, we used probes from Mauna Kea Technologies, Paris, France, to examine tissue samples. The tip of the probe was placed gently on the tissue and a sequence of images was taken. After imaging, human tissue samples were stored in 4% formalin and transferred for histopathology. Preliminary tests showed that neither the fixation process nor the age of the sample or the preoperative administration of 5-ALA has an effect on CLE examination after topical application of AF.  Figure 3 shows examples of typical brain tumor imaging by endomicroscopy technology.

### 2.3. Intraoperative Endomicroscopic Image Classification

**Figure 4:**
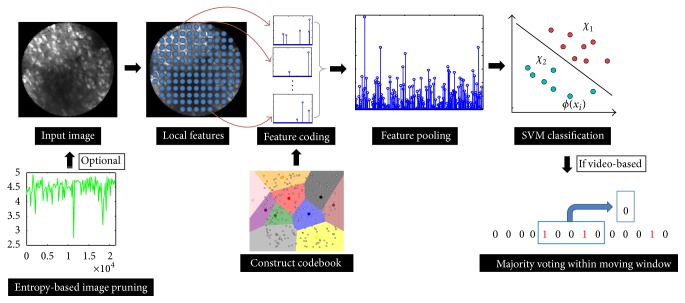
Illustration of the image recognition system for tissue classification.

Our classification pipeline includes three parts: offline unsupervised codebook learning, offline supervised classifier training, and online image and video classification. The online classification system is shown in Figure 4. The core components are local feature extraction, feature coding, feature pooling, and classification. Local feature points are detected on the input image and descriptors such as “SIFT” [7] and “HOG” [8] are extracted from each feature point. To encode local features, codebooks are learned offline. A codebook with *m* entries is applied to quantize each descriptor and generate the “code” layer. As a preferred embodiment, hierarchical *K*-means clustering method is utilized. For the supervised classification, each image is then converted into an *m*-dimensional code represented as a histogram, where each bin encodes the occurrence of a quantized feature descriptor. Finally, a classifier is trained using the coded features. As one preferred embodiment, support vector machine (SVM) [9] is utilized. Note that our system is not limited to SVM classifier. For example, as another embodiment, random forest classifier [10] can be utilized alternatively. Two variations of our system are considered. (1) If input images are considered as video streams, our system is able to incorporate the visual cues from adjacent (prior) image frames. This significantly improves the performance of our recognition system. (2) If input images are low-contrast and contain little categorical information, our system can automatically discard those images from further processing. These two variations increase the robustness of the overall system.

### 2.4. Entropy-Based Image Data Pruning

**Figure 5:**
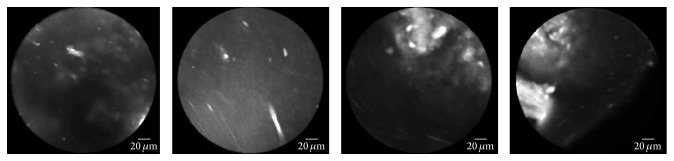
Example of excluded images due to low entropy.

Image frames with low image texture information are not clinically interesting or not discriminative for image classification task. Image entropy [11] is a quantity which is used to describe the “informativeness” of an image region, that is, the amount of information contained in a region. On the one hand, low-entropy images have very little contrast where large numbers of pixels have the same or similar intensity values. On the other hand high entropy images have a great deal of contrast from one pixel to the next. See examples of images with low information content in Figure 5.

**Figure 6:**
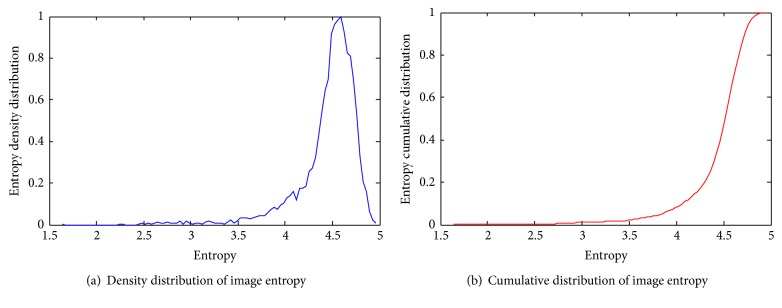
Image entropy distribution for brain tumor dataset.

To estimate a proper entropy threshold, we first calculate the distribution of the image entropy throughout the available dataset.  Figure 6 shows the image entropy distribution for brain tumor dataset. As it can be seen, there are a relatively large number of images with entropy significantly lower than that of the rest of the images within a sequence. We therefore set the entropy threshold such that 10% of images will be discarded from later stages of our system (e.g., 4.05 for brain tumor dataset).

## 3. Theory and Calculations

### 3.1. Feature Descriptor

Scale Invariant Feature Transform (SIFT) [12] is a local feature descriptor that has been used for a large number of applications in computer vision. It is invariant to translations, rotations, and scaling transformations in the image domain and robust to moderate perspective transformations and illumination variations. Experimentally, the SIFT descriptor has been proven very useful in practice for image matching and object recognition under real-world conditions.

In our system, we utilize dense SIFT descriptors of 20 × 20 pixel patches computed over a grid with spacing of 10 pixels. Dense image descriptors are necessary to capture uniform regions in cellular structures such as low-contrast regions in case of meningioma.

### 3.2. Codebook Generation

We perform hierarchical *K*-means clustering on a random subset of 100,000 local features, extracted from the training set to form a visual vocabulary. We utilized Euclidean distance based exhaustive nearest neighbor search to obtain the feature clusters. As another embodiment, one can also utilize a vocabulary tree structure (binary search tree) to obtain the clusters. For* Bag of Words* (BoW, described in the following section), we preferred the vocabulary tree structure with tree depth of 8. For* sparse coding* (SC, described in the following section) [13], and* locality-constrained sparse coding* (LLC, described in the following section) [14], we prefer *K*-means of Euclidean distance based exhaustive nearest neighbor search. The utilized vocabulary size of the codebook for our experiments is *m* and that is set to be 256. Codebook represents the most discriminative set of features, which can supposedly be used for classification. In addition, the codebook is represented within a high dimensional space defined by the number of histogram entries, that is, Bin (e.g., 256 bins). Due to high dimensional space the discrimination between the two classes may not be readily visualized.

### 3.3. Feature Coding

Let **X** be a set of *d*-dimensional local descriptors extracted from an image; that is, **X** = [*x*_1_,…, *x*_*n*_] ∈ **R** ^*d*×*n*^. Given a codebook with *m* entries, **B** = [*b*_1_,…, *b*_*m*_] ∈ **R** ^*d*×*m*^, various coding schemes have been developed to convert each descriptor into an *m*-dimensional code **c**_*i*_ = [*c*_*i*1_,…, *c*_*im*_] ∈ **R** ^*m*^. In this section, we first review three existing coding schemes (Bag of Words, sparse coding, and locality-constrained linear coding). As embodiments of our classification pipeline, these three coding schemes can be utilized in our proposed pipeline. Toward the end of this section, we will describe the proposed novel locality-constrained sparse coding method and the solution to this problem with more details.

*Bag of Words (BoW)*. As one embodiment, BoW based approach can be utilized. For a local feature *x*_*i*_, there is one and only one nonzero coding coefficient. The nonzero coding coefficient corresponds to the nearest visual word subject to a predefined distance. When we adopt the Euclidean distance, the code *c*_*i*_ is calculated as

(1) $$\begin{array}{c} c_{ij}=\left\{\begin{array}{cc} 1, & if\;\;j=\arg\underset{j=1,\ldots ,n}{\min}{\left\|x_{i}-b_{j}\right\|}_{2}^{2},\\ 0, & otherwise. \end{array}\right. \end{array}$$

*Sparse Coding*. As one embodiment, sparse coding [13] based approach can be utilized. The local feature *x*_*i*_ is represented by a linear combination of a sparse set of basis vectors in the codebook. The coefficient vector *c*_*i*_ is obtained by solving an *l*_1_-norm regularized problem:

(2a) ci=arg min xi−Bci22+λci1

(2b) s.t. 1Tci=1,∀i,

where ‖.‖_1_ denotes the *l*_1_-norm of the a vector. The constraint 1^*T*^ *c*_*i*_ = 1 follows the shift-invariant requirements of the sparse code [15].

*Locality-Constrained Linear Coding (LLC)*. Unlike sparse coding, LLC [14] enforces codebook locality instead of sparsity. This leads to smaller coefficients for basis vectors farther away from *x*_*i*_. The code *c*_*i*_ is computed by solving the following regularized least squares error:

(3a) ci=arg min xi−Bci22+λdi⊙ci22

(3b) s.t. 1Tci=1,∀i,

where ⊙ denotes the element-wise multiplication and *d*_*i*_ ∈ *R* ^*m*^ is the locality adaptor that gives different freedom for each basis vector proportional to its similarity to the input descriptor *x*_*i*_. Specifically,

(4) $$\begin{array}{c} d_{i}=\exp\frac{dist\left(x_{i},B\right)}{\sigma }, \end{array}$$

where dist(*x*_*i*_, *B*) = [dist(*x*_*i*_, *b*_1_),…,dist(*x*_*i*_, *b*_*m*_)]^*T*^, and dist(*x*_*i*_, *b*_1_) is the Euclidean distance between *x*_*i*_ and *b*_*i*_. *σ* is used for adjusting the weight decay speed for local adaptation.

*Locality-Constrained Sparse Coding (LSC)*. The proposed LSC feature coding method compares favorably to state-of-the-art methods in that it not only enforces code sparsity for better discriminative power but also preserves code locality in the sense that each descriptor is best coded within its local-coordinate system. Specifically, the LSC code can be formulated as

(5a) ci=arg min xi−Bci22+λdi⊙ci1

(5b) s.t. 1Tci=1,∀i.

To the best of our knowledge, this is the first feature coding method that takes into account both locality and sparsity in converting feature descriptor into feature code. Although various algorithms exist for solving the conventional sparse coding problem, it becomes a significantly challenging optimization problem once we incorporate the locality weight vector *d*_*i*_ as in (5a) and (5b). In the following, we present an algorithm for solving (5a) and (5b) based on Alternating Direction Method of Multipliers (ADMM) [16]. A fast approximate solver to (5a) and (5b) is also described based on *k*-NN search. We kindly point out that this is the first time such an algorithm (ADMM and its *k*-NN based fast solver) is used to solve the LSC problem.

*Optimization Algorithm*. We employ the Alternating Direction Method of Multipliers (ADMM) [16] to solve (5a) and (5b). Let us first introduce a dummy variable *y*_*i*_ ∈ *R* ^*m*^ and reformulate (5a) and (5b) as

(6a) ci=arg min xi−Bci22+λdi⊙yi1

(6b) s.t. 1Tyi=1,∀i

(6c)  ci=yi.

Then, we can form the augmented Lagrangian of the above objective, which becomes

(7) min⁡Lci,yi=xi−Byi22+λdi⊙ci1+μci−yi22+ρTci−yi+μ1T−yi22+γ1Tyi−1.

The ADMM consists of three iterations

(8a) yit+1=arg⁡minyi⁡ Lyi,cit,ρt,γt,

(8b) cit+1=arg⁡minci⁡ Lyit+1,ci,ρt,γt,

(8c) ρt+1=ρt+μci−yi,

which allows us to break our original problem into a sequence of subproblems. In subproblem (8a), we are minimizing *L*(*y*_*i*_, *c*_*i*_^*t*^, *ρ* ^*t*^, *γ* ^*t*^) with respect to only *y*_*i*_, and the *l*_1_-penalty ‖*d*_*i*_⊙*c*_*i*_‖ disappears from the objective making it a very efficient and simple least squares regression problem. In subproblem (8b), we are minimizing *L*(*y*_*i*_^*t*+1^, *c*_*i*_, *ρ* ^*t*^, *γ* ^*t*^) with respect to only *c*_*i*_, and the term ⁡‖*x*_*i*_ − **B** *y*_*i*_‖_2_^2^ + *μ*‖1^*T*^ *y*_*i*_ − 1‖_2_^2^ + *γ*(1^*T*^ *y*_*i*_ − 1) disappears allowing for *c*_*i*_ to be solved independently across each element. This now allows us to use soft-thresholding efficiently. The current estimates of *y*_*i*_ and *c*_*i*_ are then combined in subproblem (8c) to update our current estimate of the Lagrangian multipliers *ρ* and *γ*. Note that *ρ* and *γ* play a special role here, as they allow us to employ an imperfect estimate of *ρ* and *γ* when solving for both *y*_*i*_ and *c*_*i*_. For convenience, we introduce the following soft-thresholding (shrinkage) operator:

(9) $$\begin{array}{c} S_{\epsilon }\left[\;x\;\right]=\left\{\begin{array}{cc} x-\epsilon , & if\;\;x>\epsilon ,\\ x+\epsilon , & if\;\;x<-\epsilon ,\\ 0, & otherwise. \end{array}\right. \end{array}$$

*Fast Approximate Coding*. The size of the codebook *B* has a direct effect on the time complexity of algorithm described above. To develop a fast approximate solution to LSC, we can simply use the *K*  (*K* < *n*) nearest neighbors of *x*_*i*_ as the local bases *B*_*i*_ and solve a much smaller sparse reconstruction system to get the codes:

(10a) c^i=arg min xi−Bc^i22+λd^i⊙c^i1

(10b) s.t. 1Tc^i=1,∀i.

As *K* is usually very small, solving (10a) and (10b) is very fast. For searching *K*-nearest neighbors, we can apply a simple but efficient hierarchical *K*-NN search strategy. In this way, a much larger codebook can be used to improve the modelling capacity, while the computation in LSC remains fast and efficient.

*Majority Voting*. With an endomicroscopy imaging device, the clinician may obtain a real-time stream of images from the surgical field. This section describes an advanced feature of our image recognition system that can potentially reduce the classification error rate by smoothing out the classification results using a sliding time window (Figure 4). The idea is to assign class labels to the current image frame using the majority voting result of the images within a fixed length time window surrounding the current frame. To the best of our knowledge, this is the first time the majority voting scheme is used to reduce the error rate in a cellular image classification system. We experimentally verify this idea on the brain tumor dataset, and relevant results are presented in the following section.

## 4. Results

The brain tumor dataset consists of videos of two tumor categories, that is, glioblastoma and meningioma. The equipment is used to collect 86 short videos, each from a unique patient suffering from glioblastoma and 29 relatively longer videos from patients with meningioma. All videos are captured at 24 frames per second, under a resolution of 464 × 336. The collection of videos is hereafter being referred to as the brain tumor dataset. Due to the limited imaging capability of CLE devices or intrinsic properties of brain tumor tissues, the resultant images often contain little categorical information and are not useful for recognition algorithms. Image entropy has been constantly used in the literature to quantitatively determine the information content of an image. Specifically, low-entropy images have very little contrast and large runs of pixels with the same or similar values.

Uninformative video frames are discarded using entropy-based thresholding. The threshold is determined to be 4.05 after computing gray-level entropies of more than 34000 frames in our dataset. This simple thresholding scheme allows us to select more than 16 thousand frames containing glioblastoma and more than 10 thousand frames containing meningioma cases, respectively. We select 80% of these, evenly distributed over both classes, for training and the remaining 20% for testing. All experiments report average classification accuracy on 5 such training/testing splits, ensuring no frame from a training video ending up in the validation split.

**Figure 7:**
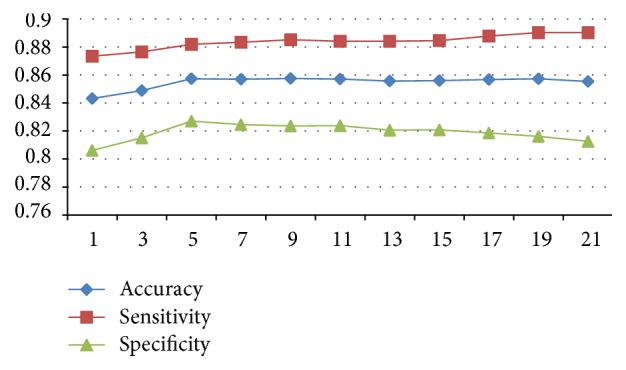
The performance of the majority voting based classification with respect to time window size.

We verify majority voting idea using the brain tumor dataset, with the same experimental setup as described in the previous sections. We set the sliding time window to be *T* in length and derive the class label for the current frame using the majority voting result of the frames within the sliding time window. The recognition performance with respect to the time window length *T* is given in Figure 7. As it can be seen the optimal performance is achieved at *T* = 5. It is quite likely that higher recognition accuracy can be achieved using much longer time window. In practice, however, one has to balance the relative importance between recognition speed and accuracy.  Table 1 depicts the results from three coding approaches including BoW, LLC, and LSC. The results include the classification metrics as well as the processing time per frame. As it can be seen form the table, the LSC method is the most accurate. However, it does come with a heavy computational cost. The future work includes improving the quality of the classification and also improving the performance of the algorithm to achieve real-time response.

## 5. Discussion and Conclusion

In this paper we have described a novel system for classifying cellular images and videos. We utilized the state-of-the-art feature coding scheme in our proposed pipeline and demonstrate that they work well on cellular image datasets. We also propose a novel feature coding method (LSC) and a novel fast solution for its implementation. As one embodiment local features are extracted densely to represent the appearance of the local image patch. However, the local feature descriptors are not limited to, for example, SIFT; various types of local feature descriptors, for example, Local Binary Pattern (LBP) [17], Histogram of Oriented Gradient (HOG) [8], and Gabor features [18], can be plugged into our pipeline easily. Future work would consist of investigating the usefulness of feature learning techniques such as Convolutional Neural Networks (CNN) [19]. Local features obtained using machine-learned filters are arguably better than hand-designed features. We expect to significantly improve the performance of our system by exploiting feature learning techniques.

## Figures and Tables

**Table 1:** The recognition accuracy and speed of different methods on the brain tumor dataset.

	Accuracy	Sensitivity	Specificity	Time (s)
BoW	0.838735	0.893285	0.771834	0.5935
LLC	0.840300	0.877257	0.794974	0.9154
LSC	0.843205	0.873402	0.806171	5.413
